# Facilitating accurate health provider directories using natural language processing

**DOI:** 10.1186/s12911-019-0788-x

**Published:** 2019-04-04

**Authors:** Matthew J. Cook, Lixia Yao, Xiaoyan Wang

**Affiliations:** 1 Center for Quantitative Medicine, University of Connecticut Health Center, Farmington, CT 06030 USA; 2 Office of the Vice President for Research, University of Connecticut, Storrs, CT 06269 USA; 3 Department of Community Medicine and Health Care, University of Connecticut Health Center, Farmington, CT 06030 USA; 4 Department of Health Sciences Research, Mayo Clinic, Rochester, MN 55905 USA; 5 Department of Family Medicine, University of Connecticut Health Center, Farmington, CT 06030 USA

## Abstract

**Background:**

Accurate information in provider directories are vital in health care including health information exchange, health benefits exchange, quality reporting, and in the reimbursement and delivery of care. Maintaining provider directory data and keeping it up to date is challenging. The objective of this study is to determine the feasibility of using natural language processing (NLP) techniques to combine disparate resources and acquire accurate information on health providers.

**Methods:**

Publically available state licensure lists in Connecticut were obtained along with National Plan and Provider Enumeration System (NPPES) public use files. Connecticut licensure lists textual information of each health professional who is licensed to practice within the state. A NLP-based system was developed based on healthcare provider taxonomy code, location, name and address information to identify textual data within the state and federal records. Qualitative and quantitative evaluation were performed, and the recall and precision were calculated.

**Results:**

We identified nurse midwives, nurse practitioners, and dentists in the State of Connecticut. The recall and precision were 0.95 and 0.93 respectively. Using the system, we were able to accurately acquire 6849 of the 7177 records of health provider directory information.

**Conclusions:**

The authors demonstrated that the NLP- based approach was effective at acquiring health provider information. Furthermore, the NLP-based system can always be applied to update information further reducing processing burdens as data changes.

## Background

**Fig. 1:**
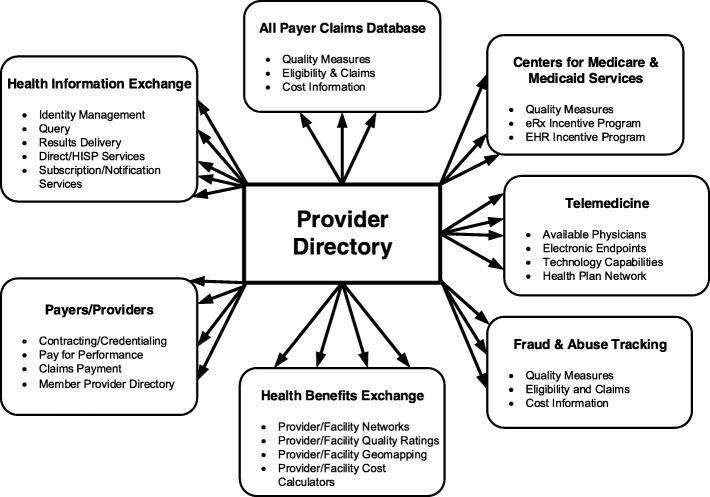
Provider directory data in health care. Arrows indicate flow of provider directory data into the various health care areas for a variety of different purposes

Accurate information of provider directory data is vital in health care. As illustrated in Fig. 1, provider directory data contain important information for many areas of health care such as health information exchange, claim databases, insurance industries [1]. When accurate information is made available, millions of individuals are empowered to make the choices that are best for themselves and their families.

Inaccurate provider directories can create a barrier to care and raise questions regarding the adequacy and validity of the health care as a whole. Accuracies of provider directories were first raised among dermatologists. It was found that among 4754 total dermatologist listings in the largest plans in 12 metropolitan areas in the United States, 45.5% represented duplicates in the same plan directory. Among the remaining unique listings, only 48.9% of dermatologists were reachable, accepted the listed plan, and offered an appointment for a fictitious patient [2].

In response to concerns over these findings, the Centers for Medicare & Medicaid Services (CMS) conducted a follow-up review of the provider directories. The CMS completed its first review round of Medicare Advantage (MA) online provider directories between February and August of 2016. This review round examined the accuracy of 108 providers’ locations selected from the online provider directories of 54 Medicare Advantage Organizations (MAOs) (representing approximately one-third of all MAOs, with 5832 providers reviewed in total). The review found that 45.1% of provider directory locations listed in these online directories were inaccurate. Within each MAO directory, the percent of inaccurate locations ranged from 1.77 to 86.53%, with an average inaccuracy rate by location of 41.37% across the MAOs reviewed. The majority of the MAOs (37/54) had between 30 and 60% inaccurate locations. Because MAO members rely on provider directories to locate an in-network provider, these inaccuracies pose a significant access to care barrier. Inaccuracies with the highest likelihood of preventing access to care were found in 38.4% of all locations [3].

Maintaining provider directory data and keeping it up to date is challenging [4]. The government and health industries expend significant resources to acquire accurate information for provider directory data. However, provider information changes quickly, and almost every piece of information contained in provider directories can become problematic. CMS report indicated that 20% of provider data changes every year. Providers may not give updated information in a timely fashion, and health industries and government may have a difficult time keeping up with frequent changes.

Federal and state regulations mandate accurate provider directories for Medicare Advantage plans or policies sold in the federally run health exchange [5–10]. State licensure lists have additional limitations in that they contain duplicate records and data on providers who may be deceased, retired, or no longer practicing in the state [6]. Furthermore, licensure lists have state specific identifiers but often lack structured national identifiers that can be used to link the information to additional information. The manual work required to acquire provider information in each of these state and federal databases, given large amount of textual information, is costly, time consuming, and difficult to keep up to date [6–8]. There is no comprehensive listing of active health professionals nationally or within states [6]. State licensure lists exist, but providers from out of state may be licensed within the state, and lists ay contain incomplete or outdated information [6, 7].

Updating provider information via credentialing is too infrequently and ineffective. Automated approaches to acquire, maintain and update provider information would be desired if possible. Natural language processing (NLP), a high throughput technology, has been applied in biomedicine for decades [11]. The NLP systems have been developed to identify, extract and facilitate large amounts of textual information through the use of automated methods that bridge the gap between unstructured text and data. NLP provides a means to transform this information into a computable form. It is expected that the automation methods available through NLP can be used to combine and code disparate textual data from state and national provider listings. NLP provides a set of computational tools and techniques for automatically extracting and combining textual information from unstructured documents. NLP has been used for facilitating retrieval of records for research [11–14], conducting biosurveillance [15–18], collecting specific data [19–21] applying clinical guidelines [22, 23], reporting quality measures [24, 25], performing clinical decision support [26–28], and coding administrative processes [29–31].

Although NLP has been used administratively to classify documents for other administrative processes, it has not been applied to process and code textual information for health providers. The objective of this study is to determine the feasibility of using NLP techniques to combine disparate resources of textual data and acquire accurate information on health providers.

## Methods

### Data

The authors obtained publically available state licensure lists of certified nurse midwives, nurse practitioners, and dentists on the Connecticut eLicensing website from the State of Connecticut Department of Public Health. The licensure lists included the name, address, and Connecticut license number of each health professional who is licensed to practice within the state. Lists are available by each type of professional license offered within the state. National individual provider information was obtained through the National Plan and Provider Enumeration System (NPPES). NPPES was created in response to the provision of HIPAA that mandated the adoption of standard unique identifiers for health care providers and health plans that electronically transmit health information. The NPPES public use files include the first and last names of providers, national provider identifier (NPI), business mailing address, provider location, phone number, gender, primary and secondary healthcare taxonomy code, and other identifiers (e.g., license, Medicare UPIN, Medicaid, and private payor plans).

### An NLP-based intelligent approach

**Fig. 2:**
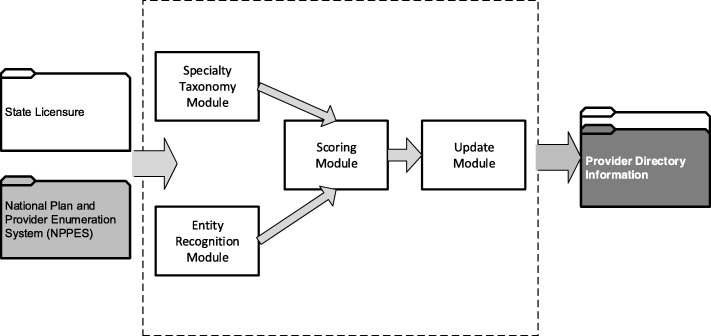
A NLP-based system to acquire and update the directory information of health providers

**Fig. 3:**
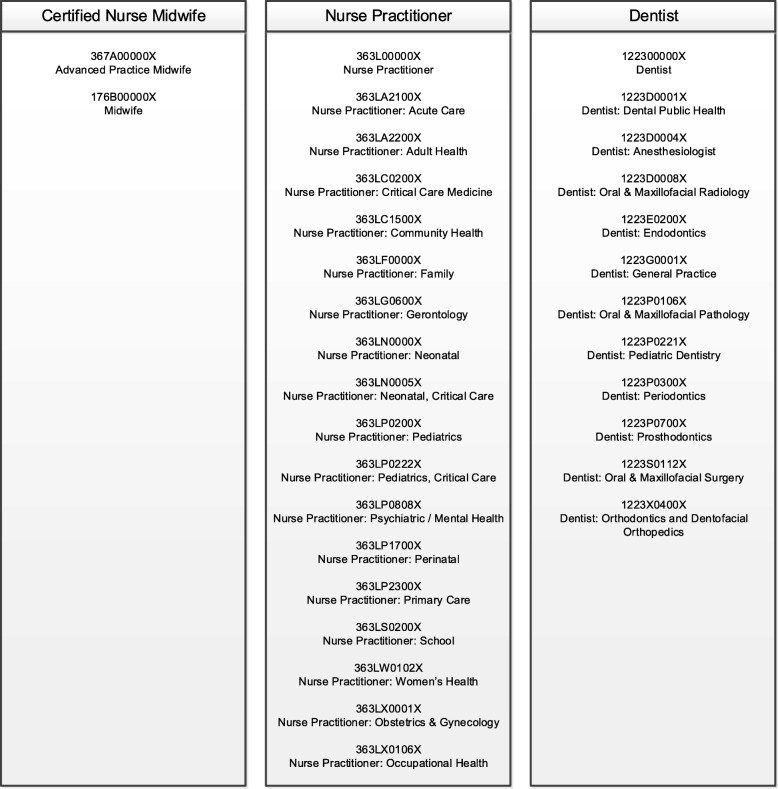
A specialty taxonomy module built to extract provider type taxonomies for certified nurse midwives, nurse practitioners, and dentists

**Fig. 4:**
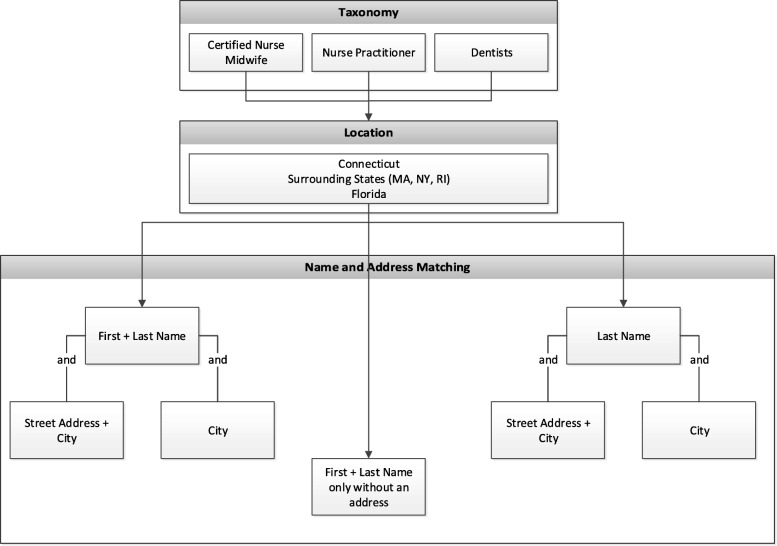
An entity recognition module implemented to acquire information on location, name and address from the disparate resources

The NLP-based system, illustrated in Fig. 2, was designed to acquire and update provider information from disparate data resources. The system contained four modules: 1) the specialty taxonomy module was implemented to obtain and match the provider taxonomies. Healthcare provider taxonomies representative of certified nurse midwifes, nurse practitioners and dentists were used in this study, as shown in Fig. 3. For example, when matching for certified nurse midwives, NPPES data were filtered to compare only healthcare provider taxonomies 367A0000X and 176B00000X for advanced practice midwife and regular midwife. Each provider type list was independently run against the NPPES for that specific taxonomy grouping. 2) The entity recognition module was used to acquire location, and name and address information to identify textual data within the state and federal records. A filter for location was applied to the NPPES database to compare only providers who were within or near Connecticut. The NLP system only included providers with addresses listed in Connecticut, states surrounding Connecticut (i.e., Massachusetts, New York, and Rhode Island), and the State of Florida (due to a high proportion of residents who reside part time in each state) to increase the likelihood that the provider would be found if he/she lived both within and out of state. Since we presumed the state licensure information would contain every licensed provider within Connecticut, those lists were used to combine with NPPES. Using NLP techniques to match records using combinations of first name, last name, street address and town. As shown in Fig. 4, seven types of name and address matches could be made for first and last name combinations with and without city and/or street address and last name only combinations with and without city and/or street address. 3) The scoring module was designed to match providers from different resources. Five years of data (2013–2017) were in the study. Four entities (last name, first name, city and street) was selected for scoring algorithms. If the records were matched between the data resources in the most recent year for an entity, the score was set to be 5 points; if not, the score will be decremented to take 1 point off for earlier years. A threshold of the points was set to determine if an accurate record was obtained. 4) The update module was employed to combine all the information from previous modules and generate the final output for accurate provider information records. The final records included legal name of the individual, national provider identifier (NPI), gender, telephone number, healthcare provider taxonomy, business practice location address, mailing address and any other identifier information that might be contained for the particular record in the NPPES database.

### Evaluation

The complete set of certified nurse midwives (231 records) was first compared to the manually labeled records serving as training set. The system was recursively tested and refined for optimal accuracy. State dentist and nurse practitioner lists were then tested I using the NLP system. A randomly selected 200 records was manually reviewed as gold standard. The performance of the NLP-based system was evaluated through an intrinsic assessment process to compare the NLP system to the gold standard reference result.

#### Quantitative evaluation

Recall and precision were used to assess the performance of our system. Recall was calculated as the ratio of the number of records that were correctly identified by the NLP system over the total number of the corresponding drug-ADE pairs in the reference standard (i.e., TP/(TP + FN)). Precision was measured as the ratio of the number of records obtained by the system that were correct according to the reference standard divided by the total number of drug-ADE pairs obtained by the NLP system (i.e., TP/(TP + FP)).

#### Qualitative evaluation

We further analyzed the records obtained by the NLP system to understand errors in the system. The types of errors was classified and summarized in the error analysis.

## Results

We identified 7408 nurse midwives, nurse practitioners, and dentists in the State of Connecticut. The initial accuracy of the NLP system was 0.82 on the training data of certified nurse midwives. After recursively refining the system, recall and precision were 0.95 and 0.93 on the test data of nurse practitioners and dentists, respectively. Using the system, we were able to accurately acquire 6849 of the 7177 records of the nurse practitioners and dentists.

The qualitative evaluation was summarized in Table 1. Qualitative evaluation indicated that challenges include providers who changed their names, moved or listed different addresses in each database, had name misspellings in either record or who had incorrect taxonomies associated with their provider information in the federal database.

**Table 1:** Types and examples of errors identified in the qualitative evaluation

Type	Example
Misspelling	Helen Black --- Hellan Black
Name Change	Helen Black --- Helen Gold
	Brown Smith --- Brown Jackson Smith
Moved to Different Addresses	1705 Park Ave, small town
	857 High Road, big town
Inaccurate Specialty Taxonomy	Advanced practice midwife --- Nurse Practitioner

## Discussion

We demonstrated that the information required for combining disparate databases is amenable to automatic extraction by the NLP system from the disparate state and federal data. The NLP algorithm performed well on obtaining accurate information for health providers. The recall and precision were 0.95 and 0.93 respectively. Using the system, we were able to accurately acquire 6849 of the 7177 records of health provider directory information.

A qualitative analysis revealed some situations that the automated process could not address. Firstly, the algorithm was not able to handle situations where health providers used their middle name as a first name in one of the databases. For example, a dentist was listed as Andrew Wang in the state licensure file, while that same provider was listed as Howard Wang in the NPPES database. Both files listed the address of 37 Collins Road. An Internet search revealed that the provider in this situation practices professionally as A. Howard Wang and doesn’t respond to his first name.

Secondly, the system could not address situations where a provider’s name has changed due to a marriage or divorce but the licensure data, most typically, was never updated to account for the new last name. This situation is more common among female health professionals. There were numerous cases when a person initially registered for licensure early in her career under a maiden name and over time that name was legally changed to another through marriage or divorce but the state licensure database listed the former last name.

Thirdly, since taxonomy was used as part of the initial filtering scheme, the NLP algorithm failed to recognize persons with the same name if the NPPES data listed them as having a primary or secondary taxonomy outside of the scope of nurse practitioner or dentist. For example, one health professional Eric Shapiro who was found on the state dentist list and practiced oral and maxillofacial surgery (i.e., healthcare provider taxonomy 1223S0112X), was found in NPPES as a physician, rather than a dentist, of maxillofacial surgery (i.e., taxonomy 204E00000X). Another situation occurred when a provider had two licenses, the first license as a nurse practitioner and another as a clinical social worker. The NPPES data only listed the social work taxonomy so this professional was not identified as a match with the licensure data as an advanced practice nurse.

The scoring module performed effectively to obtain the accurate information even one or more entities (i.e. first name, last name, city and street) was not correctly identified. Further development of the NLP-based system will address these issues by refining the rules and employing statistical approaches to assess accuracies of obtained records. However, the automated processes may never address all the human errors that occur when outdated information remains in the source data.

This study has a few key limitations. One limitation with the approach is that taxonomies might not prove to be useful in filtering lists of physicians, the remaining group of health professionals who may be eligible for the incentive program, since most health professionals are physicians and the largest group of taxonomy classes are reserved for this provider group. Removing other taxonomies for physician assistants, nurses, chiropractors, and others may not sufficiently provide enough specificity for this large group of professionals. This limitation would need to be tested further to determine the usefulness of using taxonomies for physician health professionals within the NLP system. Another limitation is that this study focused solely on provider data from Connecticut, which may not be representative of provider data in other states. Therefore, further evaluation is necessary to assess the generalizability of the approach on provider lists from other states.

## Conclusions

We found that natural language processing is a feasible approach to combine disparate data sources (i.e. state, federal or industrial sources) to obtain accurate provider directory information. The NLP-based approach can accurately identify provider information efficiently and reduce labor required to acquire accurate records by hand. The automated procedures did not, however, eliminate all manual labor. Furthermore, as data changes, the NLP-based system can always be applied to update information further reducing processing burdens.

## Abbreviations

CMS: Centers for Medicare & Medicaid Services
MA: Medicare Advantage (MA)
MAOs: Medicare Advantage Organizations
NLP: Natural language processing
NPI: National provider identifier
NPPES: National Plan and Provider Enumeration System
